# Supplementary figures and images for: Long-Read Metagenomics Improves the Recovery of Viral Diversity from Complex Natural Marine Samples
Source: mSystems. 2022 Jun 13;7(3):e00192-22. doi: 10.1128/msystems.00192-22 (PMC9238414; doi:10.1128/msystems.00192-22)

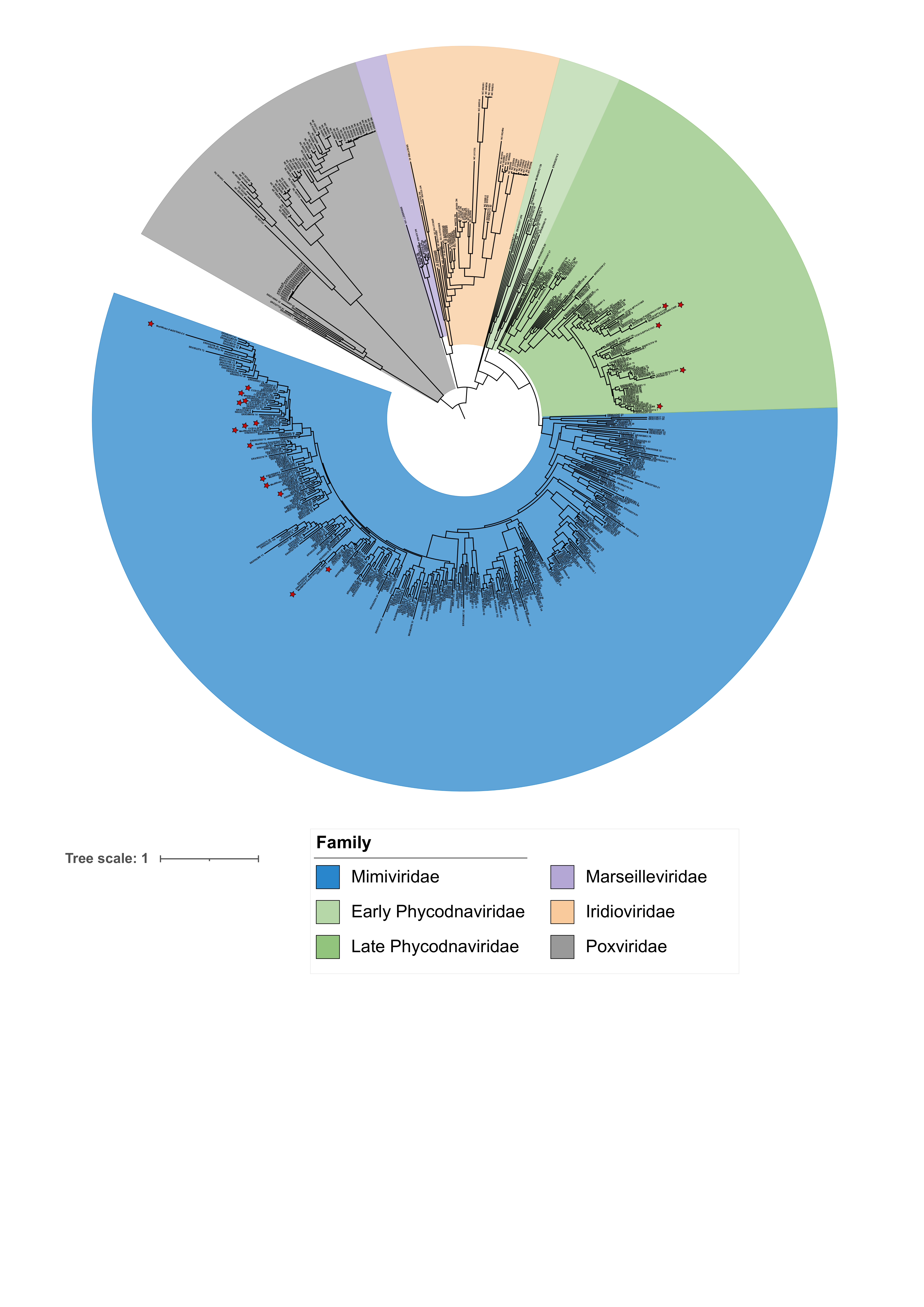

Supplement: FIG S1 [file msystems.00192-22-sf001.tif]

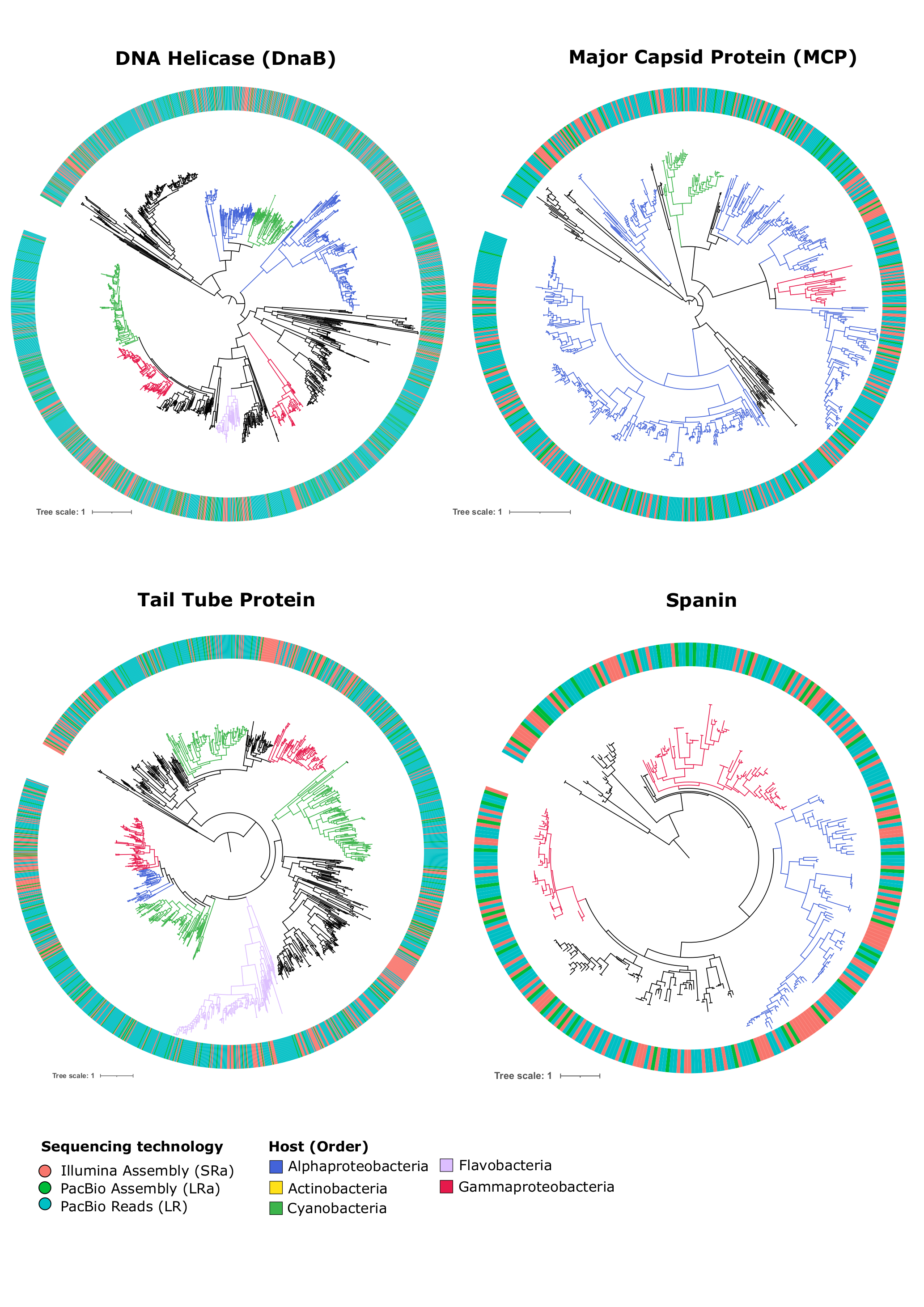

Supplement: FIG S2 [file msystems.00192-22-sf002.tif]

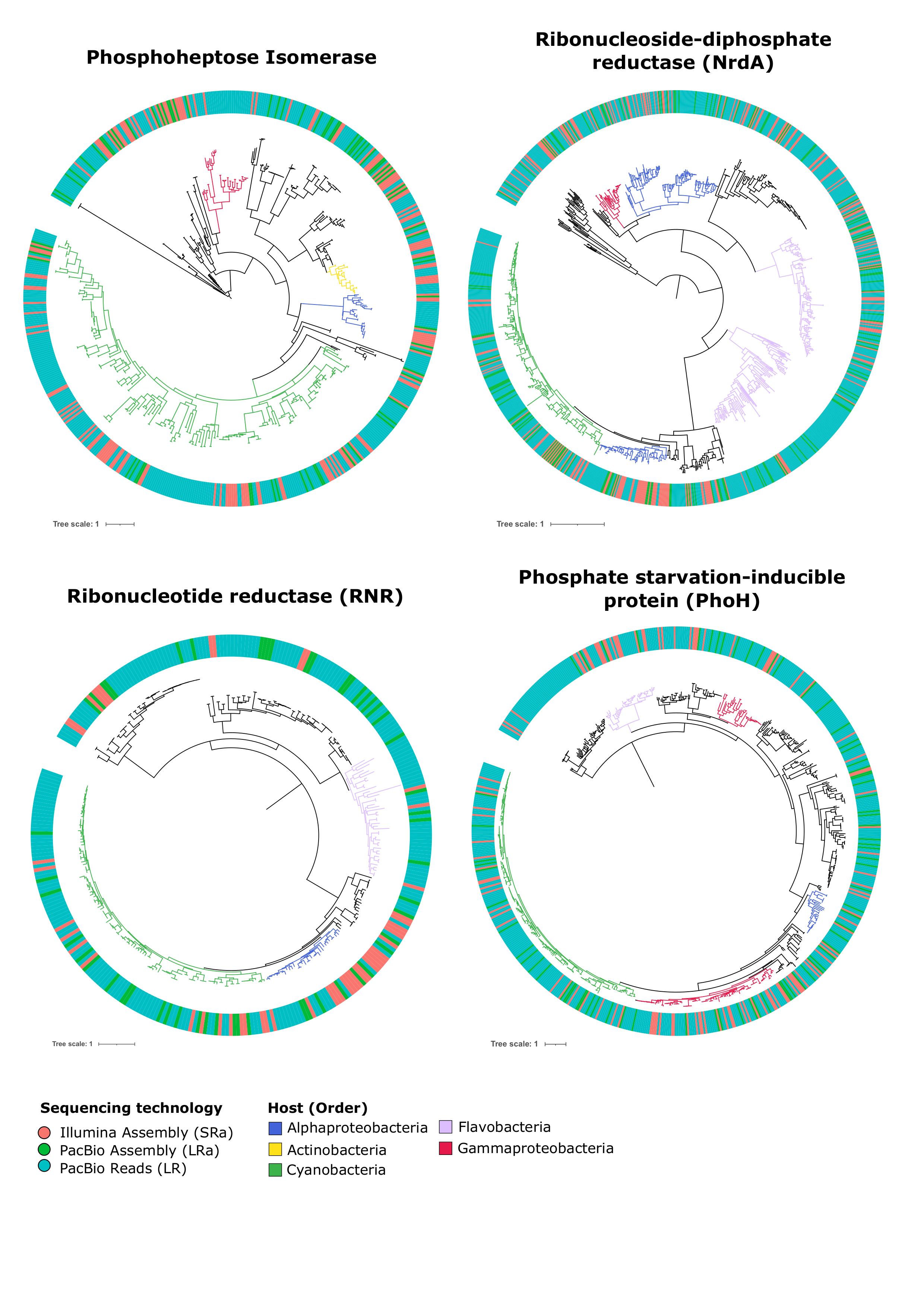

Supplement: FIG S3 [file msystems.00192-22-sf003.tif]

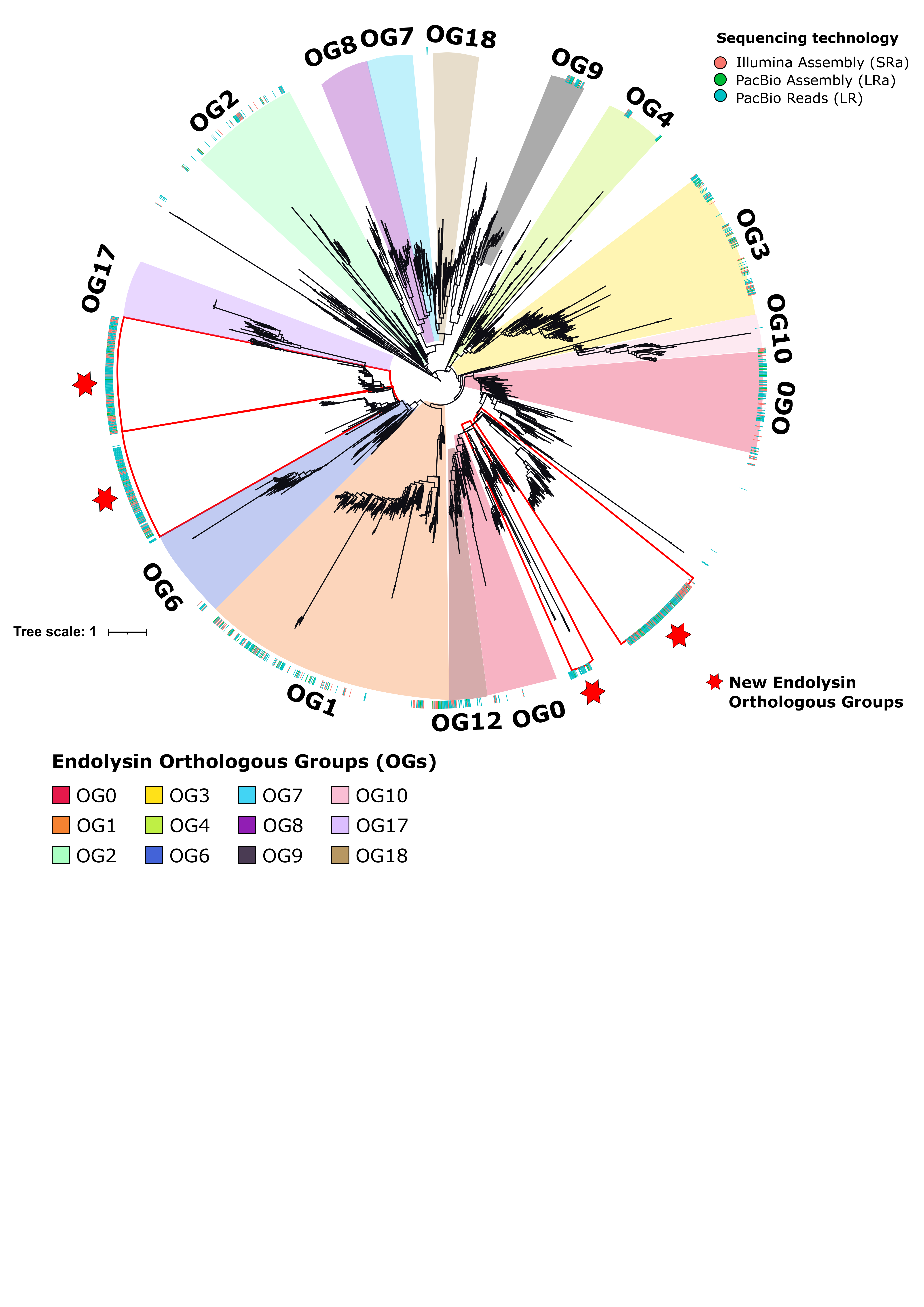

Supplement: FIG S4 [file msystems.00192-22-sf004.tif]
